# Antibacterial effectiveness of novel polyherbal gel, chlorhexidine gluconate, and calcium hydroxide as intracanal medicaments in infected root canals using real-time PCR: a randomized controlled trial

**DOI:** 10.3389/fdmed.2026.1820395

**Published:** 2026-06-03

**Authors:** Anil V. Ankola, Vinuta Hampiholi, Ram Surath Kumar, Manohar S. Kugaji, Roopali M. Sankeshwari, Varkey Nadakkavukaran Santhosh, Atrey J. Pai Khot, Kavitha Ragu, Mohammed Mustafa, Ahmed A. Almokhatieb

**Affiliations:** 1 Department of Public Health Dentistry, KLE Vishwanath Katti Institute of Dental Sciences, KLE Academy of Higher Education and Research, Belagavi, India; 2 Department of Periodontics, KLE Vishwanath Katti Institute of Dental Sciences, KLE Academy of Higher Education and Research, Belagavi, India; 3 Centre for Advanced Medical Research (CAMR), BLDE (Deemed to be University), Bangaramma Sajjan Campus, Vijaypura, India; 4 Department of Conservative Dental Sciences, College of Dentistry, Prince Sattam bin Abdulaziz University, Al-Kharj, Saudi Arabia

**Keywords:** anti-Bacterial agents, calcium hydroxide, chlorhexidine, *enterococcus faecalis*, herbal, intracanal medication

## Abstract

**Purpose:**

This study aimed to evaluate the antibacterial effectiveness of three intracanal medicaments: *Achyranthes aspera* and *Trachyspermum ammi* based novel polyherbal gel, chlorhexidine gluconate gel, and calcium hydroxide paste, against *Enterococcus faecalis* in infected root canals by means of Real-Time Polymerase Chain Reaction (qPCR) for bacterial quantification.

**Methods:**

A triple-arm, randomized controlled trial was conducted on 45 permanent mandibular and maxillary incisor and canine teeth diagnosed with asymptomatic apical periodontitis. Participants were randomly divided into three equal groups (*n* = 15), each receiving a different intracanal medicament. Microbiological samples were collected using sterile absorbent paper points at two time points: before instrumentation and after 7-day of intracanal medicament placement. The intracanal medicaments tested were a novel polyherbal gel, 1% (w/w) chlorhexidine gluconate gel, and calcium hydroxide paste. Total DNA of *Enterococcus faecalis* was extracted from these samples, and qPCR was performed to determine the bacterial quantification at both intervals. The primary outcome was the reduction in *Enterococcus faecalis* load, indicating the antibacterial efficacy of each medicament.

**Results:**

All the groups showed a significant reduction in *Enterococcus faecalis* counts from baseline to day 7. However, the chlorhexidine gel and calcium hydroxide paste exhibited significantly greater reductions than the polyherbal gel (*p* < 0.05).

**Conclusions:**

All three intracanal medicaments tested were effective in significantly reducing *Enterococcus faecalis* counts. However, the chlorhexidine gel and calcium hydroxide paste demonstrated significantly greater reductions compared to the polyherbal gel. For routine endodontic practice, chlorhexidine gel and calcium hydroxide remain more reliable choices for intracanal disinfection against *Enterococcus faecalis* and should be preferred in cases with high microbial load. The polyherbal gel, while antibacterial, may be better suited as an adjunct rather than a primary medicament in endodontic disinfection protocols.

**Trial registration:**

[https://ctri.nic.in/Clinicaltrials/pmaindet2.php?EncHid=NjU0MzY=] Clinical Trials Registry-India with registration number: [CTRI/2022/07/043862].

## Introduction

The success of root canal treatment (RCT) largely depends on the complete removal of microorganisms from the root canal system (1). However, this is often hindered by anatomical complexities, such as dentinal tubules, lateral canals, and isthmuses (2). Effective disinfection requires a combination of biomechanical instrumentation and the use of root canal irrigants and intracanal medicaments (ICMs) (3).

*Enterococcus faecalis* (*E. faecalis*)*,* a facultative anaerobic gram-positive coccus, is a key pathogen associated with persistent endodontic infections and treatment failure (4). It is commonly found in post-treatment apical periodontitis, with prevalence rates ranging from 29% to 77% in failed cases due to its antibiotic resistance (5). *E. faecalis* is the predominant species found in persistent infections and is notably resistant to conventional antibiotics (6). *E. faecalis* is highly resilient, it survives under nutrient-deprived conditions, invades dentinal tubules, tolerates high pH, and resists conventional disinfection due to adaptive mechanisms like proton pump activation (7).

Chlorhexidine gluconate (CHX) and calcium hydroxide [Ca(OH)₂] are widely used ICMs due to their known antimicrobial properties. CHX exhibits broad-spectrum antibacterial activity and substantivity but can be cytotoxic at higher concentrations (8). CHX at low concentration is widely used as an ICM because it offers a balance between effective antimicrobial action and acceptable biocompatibility. Higher concentrations of CHX can be cytotoxic to periapical tissues. However, CHX still has limitations, including its inability to fully eradicate resistant pathogens like *E. faecalis*, and potential staining and allergic reactions with prolonged use. Ca(OH)₂, another commonly used ICM with its high pH (12.5), which disrupts bacterial cell membranes and proteins (9). Despite this, it has limited effect against *E. faecalis* and has limited penetration into dentinal tubules, along with potential adverse effects such as allergic reactions and tissue irritation (10). Notably, neither CHX nor Ca(OH)₂ can consistently eliminate resistant pathogens like *E. faecalis* (11).

In light of these limitations, there is growing interest in herbal alternatives due to their potent antimicrobial and anti-inflammatory potential, enhanced biocompatibility and reduced cytotoxicity, affordability, and minimal adverse effects (12). Among these, *Achyranthes aspera* (*A. aspera*) and *Trachyspermum ammi* (*T. ammi*) demonstrated potent antibacterial and anti-inflammatory properties in traditional medicine systems (13–16). A recent *in vitro* study demonstrated significant antibacterial activity against *E. faecalis* and favourable biocompatibility (17).

Though there are reports in the literature to testify to the efficacy of various herbal ICMs for dental applications, there is little clinical evidence evaluating the antibacterial efficacy of herbal ICMs, particularly in adult permanent teeth affected by apical periodontitis. This gap underscores the need for well-designed clinical trials to assess such alternatives in real-world endodontic settings. Therefore, this study aimed to clinically evaluate and compare the antimicrobial effectiveness of a novel polyherbal gel (containing *A. aspera* and *T. ammi*), Ca(OH)₂ paste, and CHX gel in reducing *E. faecalis* load in infected mature anterior teeth. The alternative hypothesis was that the three tested ICMs would demonstrate significantly different levels of antibacterial activity over a 7-day intracanal application period.

## Methods

### Trial design and ethical considerations

This was a triple-blind, parallel-arm, randomized controlled clinical trial conducted between January 2023 and May 2023. Preferred Reporting Items for Randomized Trials in Endodontics (PRIRATE) 2020 guidelines were followed (18). Ethical approval was obtained from the Institutional Research and Ethics Committee (Ref. No: 1511). The study adhered to the principles of the Helsinki Declaration (2000). It was prospectively registered in the Clinical Trials Registry-India (Ref no: CTRI/2022/07/043862; available at: https://ctri.nic.in/Clinicaltrials/pmaindet2.php?EncHid=NjU0MzY). The study also complied with the guidelines for herbal intracanal medicaments in endodontic trials, as per the Drugs and Cosmetics Act (Gazette of India) (19).

### Study participants and sample size calculation

A total of 45 patients (age range of 17–59 years) with permanent incisor or canine teeth diagnosed with asymptomatic apical periodontitis were included after obtaining written informed consent. The inclusion criteria comprised: radiographic and clinical confirmation of apical periodontitis due to asymptomatic pulp necrosis confirmed by Digitest electrical pulp vitality test (Parkell, Edgewood, NY, USA), single-rooted permanent teeth with Vertucci Type I canal anatomy (20), apical periodontitis lesions 2–5 mm in diameter, pocket depth ≤ 3 mm, no previous RCT, and use periapical index by Orstavik (21).

The exclusion criteria included immunocompromised patients with systemic conditions; those who received antimicrobial medications over the past 3-month; teeth with extensive caries that cannot be restored; and presence of root resorption, root fractures, or canal calcification.

The sample size was determined using the GPower tool (G*Power Version 3.1.9.4 statistical software). Based on data from a previous study by Savetha et al. (22), a minimum of 15 participants per group was determined to be necessary. A total sample size of 45 was considered, considering an expected dropout rate of 10%. Power and alpha error were set at 90% and 5%, respectively.

### Randomization and blinding

**Figure 1:**
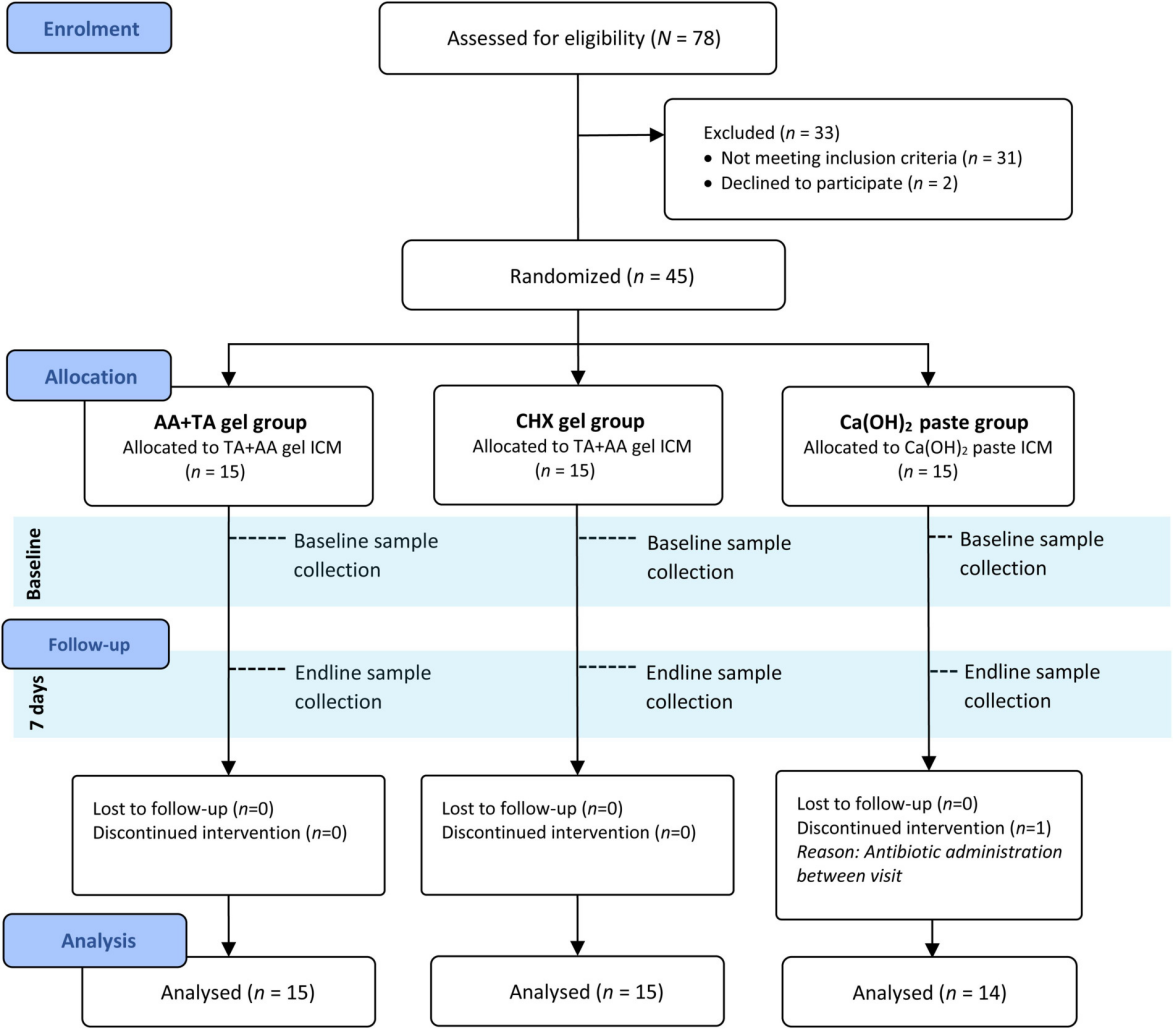
Figure depicting the methodology adopted for conducting clinical trial. AA + TA: Herbal gel formulation as an intracanal medicament containing *Achyranthes aspera* and *Trachyspermum ammi*; Ca(OH)_2_: Calcium Hydroxide paste; CHX: 1% Chlorhexidine gluconate gel.

The first allocator (A.A.) performed the clinical and radiographic diagnosis of the patients and assigned numbers based on their order of arrival at the department. A total of 45 patients (15 for each group) were then selected according to the inclusion criteria. Random codes were generated and concealed from the operator (R.K.), who carried out all RCT procedures in a blinded manner. The second allocator (V.H.) was responsible for allocating the ICMs in accordance with a randomly generated sequence created using Microsoft Excel. V.H. was not involved in patient examination, selection, or RCT. Sequentially Numbered Opaque Sealed Envelopes (SNOSE) were used to conceal the assignment sequence, with each opaque envelope labeled with the corresponding patient number. At the time of treatment, the ICMs were placed into the canals according to the random codes provided by the first allocator. The prepared canals were aseptically filled with the assigned ICMs by the second allocator (V.H.), ensuring that the treating operator (R.K.) remained blinded to both the type of ICM and the patient group. The patients were blinded to the specific ICM used. A microbiologist (M.K.) evaluated the microbiological outcomes and was also blinded to the ICM intervention. The medicaments were coded as: Syringe A: Polyherbal gel (AA + TA); Syringe B: 1% w/w CHX gel (Hexigel, ICPA Health Products Ltd., Mumbai, India); and Syringe C: Ca(OH)_2_ paste (ApexCal, Ivoclar Vivadent, Liechtenstein) with enclosed tips. Figure 1 illustrates the CONSORT flow diagram.

### Clinical procedures

Patients were instructed to rinse their mouths with 0.2% CHX mouthwash (Hexidine, ICPA Health Products Ltd., Mumbai, India) followed by the administration of 1.8 mL of local anesthetic solution comprising 1:80,000 adrenaline and 2% lignocaine hydrochloride (Lidayn, Global Dent Aids Pvt Ltd., New Delhi, India). The RCT procedure was carried out in a strict aseptic condition, and rubber dam isolation was applied. The operative field including the tooth, rubber dam sheet (Coltène Whaledent Pvt. Ltd., Panvel, India), clamp, and surroundings, was disinfected with 2.5% sodium hypochlorite (NaOCl). Carious coronal structures were removed with the aid of a sterile round bur. Access cavity was prepared under aseptic conditions using sterile round and fissure burs (Mani Inc., Tochigi, Japan). Working length (WL) was established using an electronic apex locator (E-PEX Pro, Eighteenth, Changzhou, China). Radiographic confirmation was additionally performed for all cases to verify the electronically determined WL before final instrumentation.

A 23-gauge needle and a disposable syringe (Unolok, Hindustan Syringes and Medical Devices Ltd., India) filled with sterile saline were used to flush the canal. Initial microbiological sampling was performed using 3 sterile paper points (Dentsply Maillefer, Ballaigues, Switzerland) placed to the WL for 60 s each, while pumping movements were made to extract the bacterial suspension from the main canal. The intracanal samples were transferred into tubes containing Tris-EDTA (TE) buffer. Following the manufacturer's directions, the root canals were shaped using the ProTaper Universal rotary file system (Dentsply Maillefer). The canals were then irrigated with 2 mL of 2.5% NaOCl, followed by 5 mL of saline for the final irrigant. After completing biomechanical preparation with the F2 ProTaper file, a size 25 K-file (Mani Inc., Japan) was inserted to assess apical binding and confirm optimal preparation. If no binding occurred with the size 25 K-file, preparation was extended using the F3 ProTaper file. Biomechanical preparation was completed in the same visit. Canals were dried with sterile paper points.

The tested ICMs were subsequently introduced into the canals by the second allocator (V.H.), up to 1 mm short of the WL. The amount of ICM used in each group was the same. About 1 mL of the respective tested ICM was introduced into the canal space in each case. The medicament was delivered using gentle, controlled pressure to ensure complete filling of the main canal without extrusion beyond the apex. The canal orifices were sealed with a sterile cotton pellet, and the access cavity was temporarily restored with glass ionomer cement (Fuji II, GC Corp, Tokyo, Japan). Glass ionomer cement (Fuji II, GC Corp, Tokyo, Japan) was used as an interim restoration to seal the access cavities. Patients were called back after 7-day.

At recall, the interim restorations were removed, and the operative field was disinfected as per the protocol as before. The ICMs were removed, instrumented mechanically using gentle filling using the K-file after being irrigated with 5 mL of sterile saline. Final microbiological sampling was taken as described earlier. After removal of the ICM and final sampling, root canals were obturated using gutta-percha and Apexit Plus calcium hydroxide-based root canal sealer (Ivoclar Vivadent AG, Schaan, Liechtenstein). Root canals were then obturated by means of the cold lateral compaction method. The operator (R.K.), patients, microbiologist (M.K.), and the bio-statistician were blinded to the contents of the tested ICMs and the group allocation.

### Formulation of herbal intracanal medicament

Fresh roots of *A. aspera* and seeds of *T. ammi* were obtained from the Ayurveda Pharmacy of a recognized institute in Belagavi, India. Authentication of the specimens was performed by a taxonomist from the Indian Council of Medical Research – National Institute of Traditional Medicine, Belagavi. *A. aspera* roots and *T. ammi* seeds were shade-dried and ground into a coarse powder. Ethanol extracts of both plants were prepared using a Soxhlet apparatus with 600 mL of solvent at 50 °C. The extraction cycle duration was 8 h for *A. aspera* and 5.5 h for *T. ammi*, repeated until the solvent changed from coloured to colourless. Each extraction used 150 g of coarse powder with a 1:4 powder-to-solvent ratio, producing 13.9 g (9.3% yield) of *A. aspera* and 30.2 g (20.1% yield) of *T. ammi* crude extracts. The extracts were concentrated using a rotary flash evaporator. The polyherbal ICM was prepared using weighed proportions of 5% *A. aspera* extract and 5% *T. ammi* extract (w/v), mixed with 2% glycerine. Preservatives [methylparaben (0.5%), ethylparaben (0.01%), and sodium benzoate (0.5%)] were dissolved in 4 mL of deionized distilled water. Sodium carboxymethylcellulose (2.5%) was then added and allowed to hydrate for 24 h, producing 5 mL of gel formulation. The gel was aseptically loaded into sterile syringes for direct delivery into the root canal, allowing precise placement similar to conventional ICMs. Detailed laboratory investigations on its physicochemical properties, phytochemical characterization, antibacterial and cytotoxic potency have been published by the authors Kumar RS et al. (17).

### Study outcome

The primary outcome was the reduction in bacterial load (*E. faecalis*) from baseline to post-treatment, after usage of ICM containing AA + TA gel, CHX gel, or Ca(OH)_2_ paste as determined by means of qPCR.

### qPCR for bacterial load determination

#### DNA extraction

Root canal samples were subjected to DNA extraction using the modified proteinase K method (23). After being vortexed to homogenize the samples in 1 mL of TE buffer, they were washed two to three times with fresh Tris-EDTA buffer. Subsequently, bacterial cells were lysed by using lysis buffer I and II reagents. Proteinase K (10 mg/mL) was used to degrade the protein. Samples were incubated at 60 °C for 2 h, heat-inactivated, and centrifuged. Absolute ethanol and 3M sodium acetate were used to purify the DNA-containing supernatant, which was then stored at −20 °C till it was processed further.

#### qPCR analysis

qPCR was conducted using specific primers that focus on the 16S rRNA region of *E. faecalis,* as per the recommendation given by Nandakumar et al. (24). PCR amplification was performed in a 20 μL reaction volume comprising 2 μL of template DNA, 1 μL each of specific primers (Bioserve Biotechnologies Pvt. Ltd, India), and 10 μL of TB Green Premix Ex Taq (Tli RNaseH+) (RR820A, Takara Bio inc., Japan). The mastermix included a dNTP-mixture, TaKaRa-Ex-Taq-HS, Tli-RNase-H, Mg^2^⁺, and TB-Green dye. The specific primers targeting *E. faecalis* were used. PCR reactions were carried out using a Realplex mastercycler (Eppendorf, Germany). The qPCR efficiency was calculated for serially diluted standard DNA samples of *E. faecalis* ATCC 29212, and it was found to be 105%. Table 1 summarizes the primers utilized in PCR amplification and the annealing temperatures.

**Table 1:** Information on the primers utilized in PCR amplification, including the target band size and the corresponding annealing temperatures.

Target genes and primer sequences	Thermal cycling conditions				Amplification length (bp)
	Initial denaturation	40 Cycles			
		Denaturation	Annealing	Extension	
***E. faecalis***					
F: 5ʹ-GTT TAT GCC GCA TGG CAT AAG AG-3ʹ	95℃ for 30s	95℃ for 20s	60℃ for 30s	72℃ for 30s	310 bp
R: 5ʹ-CCG TCA GGG GAC GTT CAG-3′					

F, forward primer; R, reverse primer; s, seconds; bp, basepairs.

Cycling was performed using the following protocol: an initial denaturation step followed by 40 cycles of thermal cycling conditions as part of the qPCR procedure. To guarantee the specificity of the amplification process, melting curve analysis (dissociation curve) was also conducted in the temperature range of 60 °C to 95 °C. Quantification was carried out using serially diluted DNA samples derived from the standard strain *E. faecalis* ATCC-29212 (Hi-media Laboratories Pvt. Ltd., Mumbai, India). A standard curve (Ct values versus quantity) was created using the cycle threshold (Ct) values of serially diluted DNA samples from standard *E. faecalis* strains (10⁵-10^2^ CFU/mL). Precise quantification of *E. faecalis* in the test samples was achieved by plotting the Ct values of the stored, unidentified extracted DNA against this standard curve

### Statistical analysis

Statistical analysis was performed using SPSS software (IBM Corp., Released 2012, Version 25.0, Armonk, NY, USA) statistical software. Shapiro–Wilk test was used to assess the normality of data distribution. Categorical variables were presented as frequencies with corresponding percentages, while skewed distributed numerical data were reported as medians with interquartile ranges (IQR). Group comparisons for categorical variables were made using the Chi-square test, and the Kruskal–Wallis test was applied for comparing medians across groups. The Wilcoxon signed-rank test was utilized to evaluate within-group changes over time. All statistical tests were two-tailed, with a significance threshold set at *p* ≤ .05.

## Results

### Population characteristics

**Figure 2:**
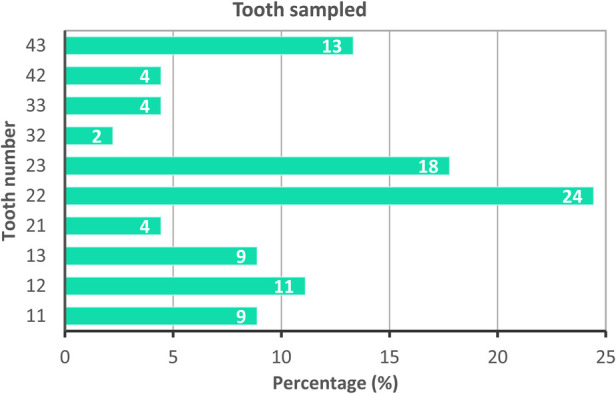
Distribution of the tooth samples based on tooth number in percentage.

Among the 78 patients evaluated, 45 were eligible and randomized (Refer to Figure 1). Only one patient (2.2%) in the Ca(OH)₂ paste group was lost to follow-up, leaving a total of 44 patients (97.8%) who were evaluated at the 7-day follow-up. The patient demographic characteristics are presented in Figure 2, Tables 2, 3. No statistically significant differences were observed in the demographic variables, including gender (*p* = .537), age (*p* = .489), and the type of tooth sampled (*p* = .240).

**Table 2:** Distribution of patients according to age and gender.

Demographic characteristics		AA + TA gel (*n* = 15)	CHX gel (*n* = 15)	Ca(OH)_2_ paste (*n* = 15)	*p*-value
**Age in years^a^**	Mean age ± SD	37.2 ± 10.3	33.2 ± 11.5	32.7 ± 13.0	0.489
**Gender^b^**	Male (%)	8 (53.3%)	6 (40.0%)	9 (60.0%)	0.537
	Female (5)	7 (46.7%)	7 (46.7%)	7 (46.7%)	

All values are expressed as mean ± standard deviation (SD) for continuous variables and as the frequency with percentages for categorical variables. The statistical test used: ^a^One-way ANOVA and ^b^Chi-square test; level of significance: *p* ≤ 0.05 is considered statistically significant.

AA + TA, Herbal gel formulation as an intracanal medicament containing *Achyranthes aspera* and *Trachyspermum ammi*; Ca(OH)_2_, Calcium Hydroxide paste; CHX, 1% Chlorhexidine gel.

**Table 3:** Distribution of tooth sampled based on tooth number.

Tooth sampled (Tooth number)	AA + TA gel (*n*)	CHX gel (*n*)	Ca(OH)_2_ paste (*n*)	*p*-value
#11	0	1	3	0.240
#12	2	0	3	
#13	2	1	1	
#21	0	0	2	
#22	4	5	2	
#23	4	3	1	
#32	1	0	0	
#33	0	2	0	
#42	1	1	0	
#43	1	2	3	
**Total (*N*** **=** **45)**	**15**	**15**	**15**	

The statistical test used: Chi-square test; level of significance: *p* ≤ 0.05 is considered statistically significant.

AA + TA, Herbal gel formulation as an intracanal medicament containing *Achyranthes aspera* and *Trachyspermum ammi*; Ca(OH)_2_, Calcium Hydroxide paste; CHX, 1% Chlorhexidine gel.

### qPCR: for bacterial load determination

The *E. faecalis* bacterial load was expressed as the number of DNA copies per μL of sample. Table 4 summarizes the decrease in bacterial counts and the corresponding median and IQR values. At baseline, qPCR analysis revealed no statistically significant differences in *E. faecalis* counts among the three groups (*p* = .289). However, bacterial counts significantly decreased in all three groups from baseline to day 7 after the placement of the ICM (*p* = .032), indicating effective antimicrobial activity of each medicament. In all groups, the number of positive samples for *E. faecalis* decreased after the placement of ICMs for 7-day, although this reduction was not statistically significant.

**Table 4:** Comparison of total bacterial counts of *Enterococcus faecalis* between the groups at baseline and 7 days.

Group	Sample	Total bacterial counts		*p*-value^b^	Change in total bacterial counts Counts (×10^2^)
		Baseline Counts (×10^2^)	7 days Counts (×10^2^)		
AA + TA gel	Median	6.89*^α^*	6.25*^α^*	0.022*	2.15^α^
	IQR	5.97–9.67	3.90–8.88		0.70–3.93
	*n* (%)	15 (100%)	14 (93.3%)		
CHX gel	Median	10.38^α^	6.25*^β^*	0.001*	4.61^β^
	IQR	6.47–22.3	4.91–7.88		1.80–16.04
	*n* (%)	15 (100%)	14 (93.3%)		
Ca(OH)_2_ paste	Median	8.76^α^	4.26^β^	0.002*	6.21^β^
	IQR	5.77–16.95	2.22–6.21		2.93–11.48
	*n* (%)	15 (100%)	13 (86.7%)		
*p*-value^a^		0.289	0.032*		0.016*

*n* denotes the number of positive samples. Different Greek symbols (α, β) indicate a significant difference between the intracanal medicaments (in the column). The statistical test used: Dunn's *post-hoc* method following a significant ^a^Kruskal–Wallis test; ^b^Wilcoxon Sign Rank test. Level of significance: *p ≤ .05 is considered statistically significant.

AA + TA, Herbal gel formulation as an intracanal medicament containing *Achyranthes aspera* and *Trachyspermum ammi*; Ca(OH)_2_, Calcium Hydroxide paste; CHX, 1% Chlorhexidine gluconate gel (w/w); IQR, Interquartile range (Q1-Q3).

**Figure 3:**
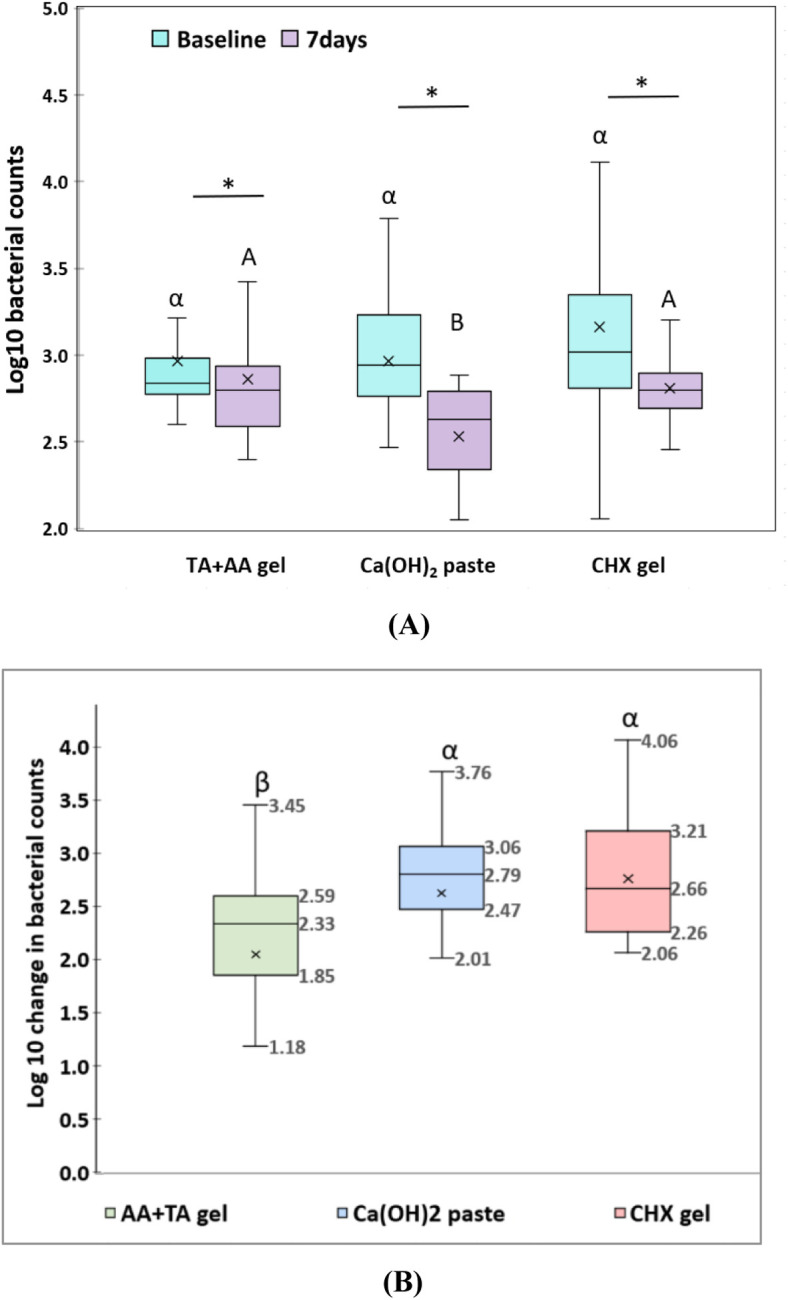
**(A)** boxplots demonstrating the statistical difference analysis for the tested ICMs, baseline and endline root canal bacteriological sample collection. Log_10_ bacterial counts of *Enterococcus faecalis* baseline and 7 days. Median values are indicated by the line within the boxplot. Different uppercase indicates a difference between the ICMs. The statistical test employed: Dunn's *post-hoc* method following a significant Kruskal–Wallis test. Statistically significant, *p* ≤ .05. **(B)** Boxplots demonstrating the Log_10_ change in bacterial counts of *Enterococcus faecalis change in baseline* and 7 days*.* Median values are indicated by the line within the boxplot. Different Greek symbols indicate a difference between the ICMs. The statistical test employed: Dunn's *post-hoc* method following a significant Kruskal–Wallis test. Statistically significant, *p* ≤ .05.

Table 4 also revealed significant differences in the extent of bacterial count reduction among the groups (*p* = .016). CHX and Ca(OH)₂ groups showed greater median reductions (4.61 × 10^3^ and 6.21 × 10^3^, respectively) compared to AA + TA group (2.15 × 10^3^). The CHX group had a significantly lower *E. faecalis* count (4.61 × 10^2^) than the AA + TA group (2.15 × 10^2^). Similarly, the Ca(OH)₂ group exhibited a significantly lower bacterial count (6.21 × 10^2^) than the AA + TA group (2.15 × 10^2^). These findings suggest that CHX and Ca(OH)₂ are more potent antibacterial agents than the AA + TA herbal gel. The log_10_-transformed bacterial counts of *E. faecalis* at baseline and after 7 days, along with the changes in these counts, are depicted in Figures 3A, 3B.

## Discussion

The present study demonstrated that all three tested ICMs resulted in a significant reduction in *E. faecalis* bacterial load after 7 days of intracanal application, as quantified by qPCR. To the best of our knowledge, this is the first clinical trial to evaluate and compare the antibacterial effects of CHX, Ca(OH)₂, and a herbal ICMs. Studies measuring microbial load reduction are necessary; however, it does not replace follow-up studies. Instead, it complements them and is equally important (25). This study acknowledges inherent variability in preoperative microbial load and apical anatomy, which are common limitations in this type of clinical research. However, the final apical preparation sizes were standardized in the present study to minimize such variations.

Advanced methods such as next-generation sequencing offer detailed identification of endodontic pathogens, but their complexity, high cost, and inter-sample variability complicate standardization (26). To balance clinical relevance with feasibility, this study focused on *E. faecalis*, a well-documented and commonly isolated organism in endodontic infections (27, 28). Its consistent detection in resistant cases makes it a suitable and reproducible target for research in endodontic microbiology (29). Molecular techniques like qPCR are more reliable than traditional culture methods, as they detect both viable and non-culturable bacteria through their genetic material. Although qPCR may detect DNA from dead cells, it remains a highly sensitive and specific tool for quantifying bacterial load (22). In this study, qPCR was effectively employed to assess the antibacterial efficacy of the three tested ICMs against *E. faecalis*, providing a robust molecular measure of treatment outcomes. Its advantages over conventional PCR, including precise quantification and reduced contamination risk, support its suitability for endodontic research (22, 30, 31).

Both CHX gel and Ca(OH)₂ paste demonstrated the highest efficacy in reducing the microbial load, followed by the AA + TA herbal ICM. This suggests that conventional chemical ICMs continue to maintain a significant advantage in terms of antibacterial effectiveness, particularly against *E. faecalis*. These findings are consistent with studies by Savitha et al. (22), Lucena et al. (32), and Önça et al. (33). In contrast, our findings differ from those of Mahfouz Omer et al. (34), who evaluated *Allium sativum*-based ICM, and Patil et al. (35), who tested herbal formulations containing mushroom, *Aloe vera*, and *Curcuma longa*. These authors reported superior antibacterial activity of herbal ICMs over Ca(OH)₂. Despite its effectiveness, CHX is associated with limitations such as dentin and restoration discoloration, limited tissue-dissolving capacity, and the formation of cytotoxic para-chloroaniline when combined with NaOCl (35). Ca(OH)₂, although widely used, has limited penetration into dentinal tubules, reduced effectiveness in the presence of debris, and prolonged use may weaken dentin, increasing tooth fracture risk (36). These drawbacks necessitate the need for careful selection of ICMs to achieve optimal root canal disinfection.

A recent *in vitro* study by Kumar et al. (17) demonstrated that a polyherbal gel formulation containing *A. aspera* and *T. ammi* for use as an ICM enhanced antibacterial effectiveness against *E. faecalis,* while maintaining good biocompatibility. These herbs were selected for their well-established antibacterial properties. *A. aspera* contains saponins and flavonoids that disrupt bacterial cell membranes and exhibit strong antimicrobial activity (37). *T. ammi* is rich in thymol and phenolic compounds, which impair bacterial enzymatic function and integrity (38, 39). The additive interaction of these components in the AA + TA gel likely contributed to the observed reduction in *E. faecalis* count, offering a promising natural alternative that does not depend on extreme pH for its activity. The specific mechanism of action and biochemical components responsible for the antibacterial activity against *E. faecalis* are unclear in *A. aspera* and *T. ammi*. In general, the antibacterial activity of *A. aspera* and *T. ammi* may primarily be attributed to the presence of flavonoids, which disrupt cytoplasmic membrane function, inhibit nucleic acid synthesis, and impair energy metabolism (40). The use of herbal ICMs such as the AA + TA gel may offer a natural and effective alternative to conventional synthetic agents, potentially improving RCT outcomes with better patient tolerance and as a suitable alternative for patients with allergies or contraindications to conventional ICMs, especially in cases involving persistent infections. Additionally, the favourable biocompatibility profile of herbal formulations may make them suitable for a broader range of patients, including those with sensitivities to chemical agents (37). Based on the study findings, the alternative hypothesis was accepted, as the three tested intracanal medicaments demonstrated significantly different levels of antibacterial activity over the 7-day intracanal application period.

One limitation of this study is the absence of an intermediate microbiological sampling after chemo-mechanical preparation and prior to ICM placement. This step is often included to distinguish the antibacterial effect of biomechanical preparation from that of the medicament. Its omission may limit the ability to attribute bacterial reduction solely to the medicaments. However, this limitation does not compromise the overall findings, as previous literature supports that instrumentation and irrigation alone significantly reduce bacterial load (41, 42). Therefore, the antibacterial effects observed over the 7-day period can still be reasonably attributed to the medicaments tested.

Future longitudinal studies should evaluate the effect of the ICM on the microhardness of root dentin and its interaction with the smear layer, and evaluate the long-term impact of such herbal ICMs on various clinical parameters, such as postoperative pain, periapical healing, long-term functional success, and patient satisfaction. A comprehensive evaluation of ICMs and exploration of their clinical effectiveness across various case scenarios, such as the inclusion of symptomatic apical and periodontitis, and incorporating polymicrobial analysis (including *Streptococcus* species).

## Conclusions

Within the study limitations, all three intracanal medicaments tested were effective in significantly reducing *E. faecalis* counts. However, CHX gel and Ca(OH)₂ paste demonstrated significantly greater antibacterial efficacy compared to the polyherbal formulation. Although polyherbal gel showed a comparatively lower reduction in bacterial counts, its effectiveness, combined with its wide availability and biocompatibility, suggests that it could still serve as a viable intracanal medicament.

## Data Availability

The data that support the findings of this study are available from the corresponding author upon request without undue reservation.
